# Depression secondary to vision loss in old age and an effective rapid screening tool for undiagnosed cases

**DOI:** 10.1186/s12991-022-00396-0

**Published:** 2022-06-02

**Authors:** Noah Clancy, Tariq Aslam, Peter Cackett

**Affiliations:** 1 University of Edinburgh Medical School, Edinburgh, UK; 2 Manchester Royal Eye Hospital, Manchester University, Manchester, UK; 3 Princess Alexandra Eye Pavilion, Chalmers Street, Edinburgh, EH3 9HA UK

**Keywords:** Vision loss, Depression, Anxiety, Macula

## Abstract

**Background:**

Zenebe et al. recently stated that despite depression being a common mental health problem in the elderly population, it is underdiagnosed in over half of the cases (Zenebe et al. in Ann Gen Psychiatry, 2021). They described an extensive list of risk factors associated with geriatric depression. However, we noted that they did not include ophthalmic conditions in this list which have previously been identified as an important risk factor for depression in the elderly.

**Main body:**

To determine the extent of undiagnosed anxiety and depression in our elderly population with vision loss, we screened a cohort of our patients, over 60 years with vision loss secondary to macular disease for both conditions.

Our cohort included 104 patients with mean best corrected visual acuity 0.58 LogMAR (Snellen equivalent 6/24). In this group, we identified 29.8% (31/104) and 28.8% (30/104) of patients with at least one depression or anxiety-related symptom, respectively, in the past 2 weeks. We identified 7.7% (8/104) and 3.8% (4/104) who had significant symptoms of depression and anxiety, respectively, that warranted further follow-up. Only two of these patients had previously been diagnosed with anxiety or depression with the majority having no previous history of either condition.

Patients from our cohort who screened for depression or anxiety often cited frustration completing tasks and loss of independence secondary to declining vision. They also complained that the vision loss resulted in a lack of confidence which in turn resulted in social isolation and loneliness. Most of the patients welcomed referral to their GP for follow-up for input regarding their mental health and they also stated an interest in attending hospital optometry low vision services and counselling support.

**Conclusions:**

With increasing time pressures on healthcare services and the rising use of virtual clinics especially during the COVID-19 pandemic, it is still essential to screen efficiently for depression in those elderly patients who are at significant risk. There is a considerable burden of major depressive disease in the geriatric population, and we would recommend that physicians (Geriatricians, GPs, Ophthalmologists etc.) screen elderly patients with vision loss for depression using the rapid screening tool which we suggest.

## Background

Zenebe et al. in their recent paper published in the Annals of General Psychiatry state that despite depression being a common mental health problem in the elderly population, it is underdiagnosed in over half of the cases [1]. In their systematic review and meta-analysis, they describe an extensive list of risk factors associated with geriatric depression, which included several co-morbidities, such as hypertension and diabetes mellitus. However, we note that they did not include ophthalmic conditions in this list which have previously been identified as an important risk factor for depression in the elderly with multifactorial mechanisms suggested relating not only vision loss, but also fear of vision loss and treatment burden [2, 3].

Mental health disorders including depression and generalised anxiety disorder have a significant burden on elderly populations with their prevalence being reported as 7.0% and 3.8%, respectively [4].

Vision loss has a high prevalence in the elderly, occurring in approximately 15% of people over 65 years [5]. It has been identified to precipitate and worsen both depression and anxiety in elderly populations resulting in a significant burden on quality of life [6]. Depression typically presents with low mood, anhedonia and fatigue, while anxiety often results in excessive worry, irritability and fatigue [7]. Furthermore, vision loss often results in patients having reduced social interaction exacerbating these disorders.

Despite research findings and supportive recommendations by the Royal College of Ophthalmologists and NICE guidelines affirming the increased population risk, screening for mental health issues is not common practice in ophthalmology clinics and this issue, therefore, deserves further attention [8]

To that end, we wanted to determine the extent of undiagnosed anxiety and depression in our elderly population attending macula ophthalmology clinics with vision loss.

## Main body

We screened a cohort of our patients, over 60 years with vision loss secondary to macular disease, attending our macula unit at the Princess Alexandra Eye Pavilion, Edinburgh for both mental health conditions. The initial screening tool comprised a patient well-being form developed at the Manchester Royal Eye Hospital to facilitate rapid screening of patients comprising both the Patient Health Questionnaire-2 (PHQ-2) and General Anxiety Disorder (GAD-2) questionnaires. Any patient scoring greater than three on the respective scoring systems were invited to complete PHQ-9 and GAD-7 screening, respectively. Significant scoring on these questionnaires would prompt a tiered referral system ranging from low vision support and GP referral to formal psychiatric review.

Our cohort included 104 patients with a mean age of 80.3 years and slight female predominance at 57.7% (60/104). The mean best corrected visual acuity of the cohort was 0.58 LogMAR (Snellen equivalent 6/24) representing moderate visual impairment. In this group, we identified 29.8% (31/104) and 28.8% (30/104) of patients with at least one depression or anxiety-related symptom, respectively, in the past 2 weeks. From those patients who were invited to complete PHQ-9 and GAD-7 questionnaires, we identified 7.7% (8/104) and 3.8% (4/104) who had significant symptoms of depression and anxiety, respectively, that warranted referral for further follow-up. Of those patients who screened positive for depression, 62.5% (5/8) had mild depression, however, of that screened positive for anxiety 75% (3/4) had moderate/severe anxiety. Only two patients in our cohort had previously been diagnosed with anxiety or depression with the majority having no previous history of either condition.

Patients from our cohort who screened for depression or anxiety often cited frustration completing tasks and loss of independence secondary to declining vision as a key driving factor for their symptoms. Patients often described their loss of independence primarily occurred after losing their ability to drive making them more dependent on friends and family. They further complained that the vision loss resulted in a lack of confidence which in turn resulted in social isolation and loneliness. Most of the patients welcomed referral to their GP for follow-up of our screening findings for input regarding their mental health. Patients also stated an interest in attending hospital optometry low vision services for provision of magnifiers and visual aids and also review by an eye clinic liaison officer for counselling support and social services input. These services directly address the burden of visual impairment on quality of life and provide support including registration of sight impairment. A combination of this visual rehabilitation and addressing the emotional effects of declining vision has been shown to be effective at improving patient symptoms [9]. Furthermore, we recommend repeat screening at follow-up appointments especially as vision loss can be dynamic and a lack of symptomatic improvement can prompt escalation to appropriate psychiatric services.

This increased risk of depression and anxiety symptoms in vision loss has also been demonstrated by Fernández-Vigo et al., using the Hospital Anxiety and Depression Scale (HADS) questionnaire as a screening method [10]. In their study, 26.9% and 25.5% of patients in their cohort had symptoms of depression and anxiety, respectively. However, this screening tool is time-consuming, taking approximately 40 min to complete and, therefore, not practical to implement into regular practice in the real world in busy clinics. Our rapid screening tool, utilising PHQ and GAD questionnaires, has additional benefits. These include ease of use and implementation, as they can be self-administered with no clinical knowledge and completed in less than 5 min provided that the patient has sufficient vision. In addition, they have successfully been demonstrated to be effective at identifying depression and anxiety, respectively [11, 12]

Our cohort only identified 2/104 patients that already had established diagnoses of depression or anxiety which is significantly lower than the expected rates given their respective prevalences. This reinforces the underdiagnosis of these conditions and there may be additional explanations for this finding. It has previously been identified that patients with mental health disorders and visual loss are less likely to seek and engage with ophthalmic services [11]. This often results in further deterioration of their vision, thereby worsening their mental health; a well-established correlation [13]. Furthermore, this reduced engagement has been exacerbated by the COVID-19 pandemic which has resulted in a significant decrease in outpatient and surgical ophthalmic services thereby lengthening waiting lists [14, 15].

With increasing time pressures on healthcare services and the rising use of virtual clinics especially during the ongoing Covid-19 pandemic, it is still essential to screen efficiently for depression and anxiety in face-to-face clinics in those elderly patients who are at significant risk. There is a significant burden of mental health conditions on the geriatric population. Major depressive disease in the geriatric population was found to result in a 65% loss in quality-adjusted life years, respectively, with early intervention facilitating better outcomes for these patients [16]. It is, therefore, essential that these patients are screened early, and treatment promptly started.

## Conclusions

Our study has confirmed the increased risk of depression and anxiety symptoms in elderly patients with visual loss secondary to macular disorders. Rapid screening techniques are effective at identifying both patients at risk and with underlying disorders and can facilitate prompt management of the conditions.

We, therefore, recommend that physicians (Geriatricians, GPs, Ophthalmologists etc.) screen elderly patients with vision loss for depression on a regular basis using the rapid screening tool which we suggest. This will hopefully reduce the problem of underdiagnosed depression in the elderly and allow appropriate action to be taken for those in need of further input and care from specialist services.

## Data Availability

The data set generated and analysed in the study is available from the corresponding author on reasonable request.

## Abbreviations

PHQ-2/9: Patient Health Questionnaire 2/9-item
GAD-2/7: Generalised Anxiety Disorder 2/7-item
LogMAR: Logarithm of the Minimum Angle of Resolution
GP: General Practitioner
HADS: Hospital Anxiety and Depression Scale
